# Integrated analysis of pain, health-related quality of life, and analgesic use in patients with metastatic castration-resistant prostate cancer treated with Radium-223

**DOI:** 10.1038/s41391-021-00412-6

**Published:** 2021-08-26

**Authors:** Sushil K. Badrising, Rebecca D. Louhanepessy, Vincent van der Noort, Jacobien Kieffer, Jules L. L. M. Coenen, Paul Hamberg, Aart Beeker, Nils Wagenaar, Marnix Lam, Filiz Celik, Olaf J. L. Loosveld, Ad Oostdijk, Hanneke Zuetenhorst, Jeantine M. de Feijter, Vincent O. Dezentjé, Suzan Ras-van Spijk, Erik Vegt, John B. Haanen, Lonneke V. van de Poll-Franse, Wilbert Zwart, Andries M. Bergman

**Affiliations:** 1 Department of Medical Oncology, Netherlands Cancer Institute, Amsterdam, The Netherlands; 2 Department of Biometrics, Netherlands Cancer Institute, Amsterdam, The Netherlands; 3 Department of Psychosocial Research and Epidemiology, Netherlands Cancer Institute, Amsterdam, The Netherlands; 4 Department of Medical Oncology, Isala, Zwolle, The Netherlands; 5 Department of Medical Oncology, Franciscus Gasthuis & Vlietland, Rotterdam, The Netherlands; 6 Department of Medical Oncology, Spaarne Gasthuis, Hoofddorp, The Netherlands; 7 Department of Nuclear Medicine, Ziekenhuisgroep Twente, Hengelo, The Netherlands; 8 Department of Nuclear Medicine, UMC Utrecht, Utrecht, The Netherlands; 9 Department of Nuclear Medicine, Deventer Hospital, Deventer, The Netherlands; 10 Department or Medical Oncology, Amphia Hospital, Breda, The Netherlands; 11 Department of Nuclear Medicine, Isala, Zwolle, The Netherlands; 12 Department of Nuclear Medicine, Netherlands Cancer Institute, Amsterdam, The Netherlands; 13 Division of Oncogenomics, Netherlands Cancer Institute, Amsterdam, The Netherlands

**Keywords:** Cancer therapy, Prostate cancer, Cancer therapy, Outcomes research

## Abstract

**Background:**

Radium-223 (Ra-223), an alpha-emitting radiopharmaceutical, established an improved overall survival and health-related quality of life (HRQoL) in symptomatic metastatic castration-resistant prostate cancer (mCRPC) patients. However, effects on pain were not specifically evaluated. Here we assess integrated HRQoL, pain, and opioid use in a contemporary, more extensively pretreated, symptomatic and asymptomatic mCRPC population.

**Methods:**

mCRPC patients scheduled for Ra-223 treatment were included and analyzed for HRQoL, pain, and opioid use, using Functional Assessment of Cancer Therapy-Prostate (FACT-P) and Brief Pain Inventory-Short Form (BPI-SF) questionnaires and recording of opioid use and dosage, respectively. Primary outcome measure was the percentage of patients experiencing a complete pain response (score of 0 on the BPI-SF Worst pain item and no increase in daily use of analgesics). A complete or partial pain response (better BPI-SF score and decrease in opioid use) and a better or no change in HRQoL was evaluated as an integrated overall clinical response (IOCR). Secondary endpoints included the time to pain progression (TPP) and Total FACT-P deterioration (TTFD), defined as time from first Ra-223 treatment to clinical meaningful increase in BPI-SF Worst pain item score and Total FACT-P score, respectively.

**Results:**

This registry included 300 patients, of whom 105 (35%) were evaluable for FACT-P and BPI-SF during Ra-223 treatment. Forty-five (43%) patients had pain at baseline (PAB) (BPI-SF Worst pain score 5–10 points) and 60 (57%) had no pain at baseline (no-PAB) (BPI-SF Worst pain score 0–4 points). Complete pain response was achieved in 31.4% of the patients, while 58% had an IOCR. The median TTP and TTFD were 5.6 and 5.7 months, respectively, while the difference between PAB and no-PAB patients was not significant.

**Conclusions:**

In contemporary, extensively pretreated mCRPC patients, Ra-223 treatment induced complete pain responses while integrated analysis of HRQoL, pain response, and opioid use demonstrated that the majority of patients derive clinical benefit.

## Introduction

Each year, over 1.2 million men are diagnosed with prostate cancer worldwide and approximately 350,000 patients succumb to the consequences of this disease, rendering it the most common non-cutaneous cancer in males and the second largest cause of cancer-related death in men [1]. Metastatic castration-resistant prostate cancer (mCRPC) is the end stage of this disease with high morbidity and mortality as hallmarks [2]. Up to 90% of mCRPC patients develop bone metastases, which are not only associated with a shorter life expectancy, but also with cancer-related pain and skeletal-related events, including pathological fractures, compression of the spinal cord, vertebral instability, and hypercalcemia, which all affect health-related quality of life (HRQoL) [3]. Symptoms and complications of bone metastases can be treated with analgesics, external beam-radiation therapy (EBRT), bisphosphonates, RANK-ligand inhibitors, surgery, and radiopharmaceuticals [4].

In the ALSYMPCA study, the alpha-emitter Radium-223 dichloride (Ra-223) showed a 3.6 month overall survival (OS) benefit and favorable HRQoL in symptomatic mCRPC patients [4]. However, the effect of Ra-223 on pain was not evaluated using pain-specific questionnaires, and changes in the dosages of analgesics were not considered in the evaluation of pain [5]. Another study showed that asymptomatic mCRPC patients treated with Ra-223 had better treatment outcomes than symptomatic patients, but HRQoL and pain were not assessed [6]. Since completion of the accrual of patients into the ALSYMPCA study, the number of treatment options for mCRPC patients has expanded significantly. Consequently, contemporary patients treated with Ra-223 are more extensively pretreated with novel agents, like abiraterone, enzalutamide, and cabazitaxel [7]. This questions the relevance of HRQoL results from the ALSYMPCA for present mCRPC patients [8]. Given the paucity of knowledge of the effect of Ra-223 on pain and HRQoL in contemporary symptomatic and asymptomatic mCRPC patients, there is a need for a re-evaluation [8].

The primary objective of this prospective observational study was to evaluate the efficacy of Ra-223 on patient-reported pain and analgesics use. Secondly, we performed an integrated analysis of the effect of Ra-223 on patient-reported pain, analgesic use, and HRQoL in a contemporary real-life cohort. Since in daily practice, both patients with pain at baseline (PAB) and no-pain at baseline (no-PAB) are treated with Ra-223, these subgroups were assessed separately [9].

## Methods

### Study population and design

A non-interventional, multicenter, prospective observational registry was initiated to evaluate clinical outcomes, HRQoL, pain, and analgesic use in a real-life mCRPC population treated with Ra-223. The study design is fully described elsewhere [8]. In short, patients with progressive mCRPC and scheduled for Ra-223 treatment were included prospectively in 20 hospitals in the Netherlands (intention-to-treat population). There were no other inclusion and exclusion criteria or stopping rules. Paper questionnaires were sent to the patients 1 week before each treatment and in monthly follow-up, which were returned by mail to the data management office. This registry was approved by local medical ethics committees. Obtaining signed informed consent for the study was not required, but patients had to provide oral consent and written approval for registration and use of their identifiers.

### Procedures

Patients were treated with Ra-223 at 4-week intervals. Dosing was according to the manufacturers’ guidelines, which was a dose of 50 kBq per kilogram of body-weight intravenously and from April 2016 on, 55 kBq per kilogram of body-weight intravenously. Number of treatments was at the physician’s discretion, who provided the motivation for discontinuation. Patients were evaluated at the outpatient clinic prior to each treatment, where performance scores and clinical lab assessments were documented. Radiological evaluation during and after Ra-223 treatment and frequency of follow-up visits were at the physician’s discretion. Patients’ baseline characteristics within 14 days prior to the first Ra-223 treatment were recorded. Baseline characteristics, efficacy assessments, and patient-reported outcome measures (PROMs) were stored in an electronic case-report form. Follow-up was continued until start of subsequent treatment or death. Data collection was performed on-site at the end of follow-up.

### Patient-reported outcome measures

HRQoL and pain were assessed using the validated PROMs, Functional Assessment of Cancer Therapy-Prostate (FACT-P) and Brief Pain Inventory-Short Form (BPI-SF), respectively [10–12]. Furthermore, patients were asked to list all analgesic drugs (free text: name, dose, frequency, and period of use) used in the previous 4 weeks. Patients were requested to complete all questionnaires at baseline and every 4 weeks during and after Ra-223 treatment until start of subsequent treatment or death. Patients were considered evaluable for pain, opioid use, and HRQoL analysis when baseline questionnaires and at least one set of questionnaires during treatment were returned. According to published algorithms, scale scores were calculated when at least 50% of the items in that scale had been completed [10–12]. An overview of the questionnaires and their use and interpretation is provided in Supplementary Table 1.

#### BPI-SF

The BPI-SF contains 4 items on pain severity (Worst pain, Least pain, Average pain, and Current pain) and 7 items on pain interference (e.g., during sleep, walking, daily activities) [10]. Every question is scored from 0 to 10, where 0 is no pain/interference and 10 is the worst imaginable pain/interference (Supplementary Table 1). The clinically meaningful change of BPI-SF score (CMC-BPI) was defined as a change of score of at least 30% from baseline, with a minimum of 2 points [10, 11]. Two groups in the cohort were separately analyzed; no-PAB patients were defined as a Worst pain score at baseline between 0 and 4 points, and PAB patients were defined as a Worst pain score between 5 and 10. This division is in line with the Initiative on Methods, Measurement and Pain Assessment in Clinical Trials (IMMPACT) recommendations [13].

#### FACT-P

The FACT-P is a validated 39-item questionnaire, including the FACT-General subscales: Physical well-being (PWB), Social/Family well-being (SWB), Emotional well-being (EWB), Functional well-being (FWB), and a prostate cancer subscale (PCS) [12]. Items are rated on a five-point scale ranging from 0 (not at all) to 4 (very much). Subscales as well as the total score can be calculated by the sum of the items. When not all subscales are evaluable, the total score cannot be calculated. The range of these scores is (0–156) for the FACT-P total score, (0–28) for the PWB, SWB, and FWB, (0–24) for EWB, and (0–48) for PCS (Supplementary Table 1). The clinically meaningful change of FACT-P (CMC-FACT) was defined as a minimal change of 10 points from baseline for the Total FACT-P, 3 points from baseline for the subscales and 2 points from baseline for pain. A higher score indicates a better HRQoL [14].

#### Analgesic use

Patients were asked to fill out a list of all analgesics, dosages, and frequencies used in the past 4 weeks (Supplementary Table 1). Dosages of the various opioid drugs and formulations were converted to oral morphine equivalents in mg per day (Supplementary Table 2). Non-opioids and on-demand opioids were not included in our analysis.

### Endpoints and statistical analyses

All endpoints were evaluated as changes in PROMs scores from baseline, meeting predefined criteria. The primary endpoint of the study was the percentage of patients experiencing a complete pain response. In line with the International Bone Metastases Consensus Working Party (IBMCWP), a complete pain response was defined as a score of 0 on the BPI-SF Worst pain item and no increase in daily use of analgesics; a partial response was defined as a pain reduction of at least 2 points on the BPI-SF Worst pain item or a reduction of at least 25% of daily use of analgesics; pain progression was defined as an increase in pain of at least 2 points on the BPI-SF Worst pain item or an increase of at least 25% of daily analgesic use. Indeterminate response was defined as all pain decreases, not captured by complete response or partial response [15]. Patients were categorized according to their best response.

Secondary endpoints included the percentage of patients experiencing a partial and an indeterminate pain response. Moreover, patients were categorized by their Total FACT-P response, which was “improved HRQoL” (better score meeting CMC-FACT), “no change in HRQoL” (no change or changes not meeting CMC-FACT), or “worse HRQoL” (deteriorated score meeting CMC-FACT). A complete or partial pain response and an improved HRQoL or no change in HRQoL were evaluated as an integrated overall clinical response (IOCR).

Moreover, secondary outcomes included time to Total FACT-P deterioration (TTFD), time to pain progression (TPP), progression-free survival (PFS), and OS. Definitions of the secondary endpoints are listed in Supplementary Table 3. All time-to-event endpoints were estimated using the Kaplan-Meier product limit method. Patients who did not experience an event of interest were censored at their last day of follow-up for OS and PFS and at the time of their last questionnaire for TTFD or TPP.

### Sample size calculation

The rationale for sample size calculation is detailed in Supplementary Text 1. In short, a sample size of at least 120 evaluable patients was required to provide statistical power of 81% to detect significant increase in proportion of pain response rate compared to the placebo rate of 20%. With an estimated PROM response rate of 40%, we aimed to include 300 patients.

#### Software

TENALEA, an online service, was used to collect data. IBM SPSS statistics for iOS, version 25 (IBM Corp. Released 2017, Version 25.0. Armonk, NY: IBM Corp.) and Statistical Analysis System (SAS) statistical software were used for statistical analysis and for constructing graphs. Additional graphs and analyses were made and performed using GraphPad Prism for iOS version 8.00, GraphPad Software, La Jolla, CA, USA, www.graphpad.com.

## Results

### Baseline characteristics and survival

Between April 2015 and March 2018, 305 mCRPC patients from 20 Dutch hospitals scheduled for Ra-223 treatment were included. Five patients were excluded because written approval to use identifiers (name, address, residence) could not be retrieved or was not stored according to guidelines (Supplementary Fig. 1). This registry included 300 patients (registry sample), of whom 121 (40%) completed the baseline questionnaires, and 105 (35%) completed baseline and at least one follow-up BPI-SF and FACT-P questionnaire and were therefore evaluable for the individual questionnaires (evaluable sample). In all, 103 patients were evaluable for pain response analysis, because 2 patients provided insufficient data on analgesics use.

The registry sample and the evaluable sample were comparable on most baseline characteristics, survival characteristics, and treatment outcomes (Table 1 and Supplementary Table 4). However, patients in the evaluable sample significantly used calcium/vitamin D supplementation more often, and bisphosphonates less often than patients in the registry sample. Moreover, evaluable patients less often received EBRT in the 12 weeks prior to Ra-223. Although there was no significant difference in PFS, OS was significantly shorter in the registry sample than in the evaluable sample (15.2 and 19.6 months, respectively, *p* = 0.04).

**Table 1:** Baseline characteristics of the registry sample and symptomatic and asymptomatic evaluable patients.

Patient demographics	Median or value [IQR], no. of patients (%)					
	Registry sample (*n* = 300)	Evaluable sample (*n* = 105)	*p*	Pain at baseline (*n* = 45)	No pain at baseline (*n* = 60)	*p*
Age, years	73 [67–78]	73 [68–77]	ns	73 [68–77]	72 [66–78]	ns
ECOG performance status, no. of patients (%)			ns			ns
0–1	264 (88.0)	94 (90)		39 (87)	55 (92)	
2	15 (5.0)	3(3)		2 (4)	1 (2)	
≥3	0	0		0	0	
Missing data	21 (7.0)	8 (8)		4 (9)	4 (7)	
Gleason score, no. of patients (%)			ns			ns
≤7	87 (29.0)	27 (26)		10 (22)	17 (28)	
8	67 (22.3)	32 (30)		12 (27)	20 (33)	
≥9	95 (31.7)	27 (26)		14 (31)	13 (22)	
Missing data	51 (17.0)	19 (18)		9 (20)	10 (17)	
Metastatic sites, no. of patients (%)						
Bone	297 (99.0)	100 (95)	ns	44 (98)	56 (93)	ns
Lymph nodes	84 (29.0)	22 (21)	ns	10 (22)	12 (20)	ns
Visceral organs	0	1 (1)	ns	0	1 (2)	ns
Missing data	3 (1)	3 (3)		0	3 (5)	
No. of bone metastases, no. of patients (%)			ns			ns
0–1	0	2 (2)		1 (2)	1 (2)	
2–6	21 (7.0)	12 (11)		5 (11)	7 (2)	
>6	246 (82.0)	87 (83)		37 (82)	50 (80)	
Super scan	5 (1.7)	2 (2)		0	2 (3.1)	
Missing data	28 (9.3)	6 (6)		3 (7)	3 (5)	
Laboratory values						
PSA, μg/l	72.3 [25.0–175.0]	72 [22–179]	ns	73 [16–225]	72.0 [23–172]	ns
Hemoglobin, mmol/l	12.6 [11.3–13.4]	12.6 [11.6–13.4]	ns	12.3 [11.6–13.4]	12.7[11.6–13.4]	ns
ALP, U/l	138 [85–248]	118 [75–242]	ns	136 [85–330]	102 [73–186]	ns
ALP ≥220 U/l, *n* (%)	81 (27.0)	28 (27)	ns	15 (33)	13 (22)	ns
LDH, U/l	225.0 [192–296]	213 [183–280]	ns	237 [190–298]	206 [179–237]	0.07
Albumin, g/l	42 [38–44]	42 [40–44]	ns	42 [39–44]	42 [40–44]	ns
Calcium, mmol/l	2.4 [2.3–2.4]	2.4 [2.3–2.4]	ns	2.3 [2.2–2.4]	2.4 [2.3–2.4]	0.06
Testosterone, nmol/l	0.5 [0.45–0.50]	0.5 [0.5–0.5]	ns	0.5 [0.5–0.5]	0.5 [0.3–0.5]	ns
Previous lines of systemic treatments (%)			ns			ns
0	34 (11.3)	10 (10)		5 (11)	5 (8)	
1	104 (34.7)	34 (32)		10 (22)	24 (40)	
2	96 (32.0)	35 (33)		21 (47)	14 (23)	
3	50 (16.7)	19 (18)		4 (9)	15 (25)	
4	13 (4.3)	5 (5)		4 (9)	1 (2)	
5	3 (1.0)	1 (1)		1 (2)	0	
Missing data	0	1 (1)		0	1 (2)	
Specific previous treatments, no. of patients (%)						
Abiraterone and or Enzalutamide	214 (71.3)	75 (71)	ns	31 (69)	44 (73)	ns
Docetaxel	197 (65.7)	73 (71)	ns	35 (78)	38 (63)	ns
Cabazitaxel	52 (17.3)	18 (17)	ns	10 (22)	8 (13)	ns
Radiotherapy 12 weeks prior to treatment	23 (8)	2 (2)	0.01	2 (4)	0	ns
Concomitant medication, no. of patients (%)						
Bisphosphonates	49 (16.7)	11 (10)	0.03	3 (7)	8 (13)	ns
Denosumab	63 (24.4)	25 (24)	ns	14 (31)	11 (18)	ns
Calcium/vitamin D	123 (41.0)	55 (52)	0.02	25 (56)	30 (50)	ns
Analgesics use		*n* = 103		*n* = 44	*n* = 59	
Non-opioids	NA	3 (2.9)		0 (0)	3 (6.7)	
Opioids	NA	38 (36)		25 (56)	13 (22)	<0.001
Dose (mg/day)^a^	NA	44.4 [18.8–111.6]		60 [15–118.8]	30 [30–75]	ns

Data are *n* (%), median or value [IQR]. *ECOG* Eastern Cooperative Oncology Group, *PSA* serum prostate-specific antigen, *ALP* serum alkaline phosphatase, *LDH* lactate dehydrogenase, *ns* not significant, *NA* not available.

^a^Oral morphine equivalent.

Of the 105 evaluable patients, the majority received Ra-223 as a third or higher line mCRPC treatment and previously received docetaxel and abiraterone or enzalutamide (Table 1). Forty-five patients had PAB and 60 had no-PAB (Supplementary Fig. 1 and Table 1). The baseline characteristics of the two groups were comparable, however, more PAB patients used opioids (51.2% and 16.7%, respectively, *p* < 0,001). After a median follow-up of the evaluable sample of 13.2 months, PAB patients had a significantly shorter OS than no-PAB patients (13.5 and 20.3 months, respectively, *p* = 0.05) (Supplementary Table 4 and Supplementary Fig. 2).

### Pain and health-related quality of life

Questionnaire completion rates per time point are listed in Supplementary Table 5.

#### BPI-SF

**Fig. 1:**
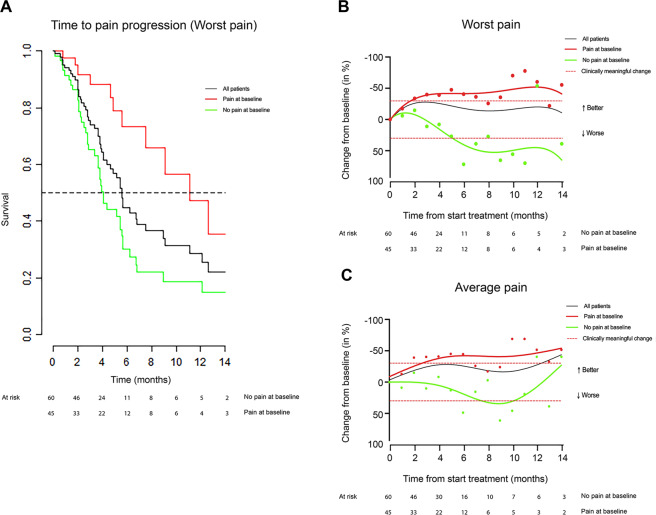
Brief Pain Inventory (BPI). **A** Kaplan-Meier estimates of time to clinically meaningful Brief Pain Inventory Short Form (BPI-SF) Worst pain subscale score deterioration for the evaluable sample (black line), patients with pain at baseline (red line), and patients without pain at baseline (green line). The horizontal dotted line represents 50% events. **B** Change in BPI-SF – Worst pain. **C** Average pain subscale scores over time in the evaluable sample (black line), patients with pain at baseline (red line), and patients without pain at baseline (green line). Data points show average scores at time points, while the lines are made to fit the trend of change of score in time. The horizontal dotted lines represent the threshold for clinically meaningful change from baseline.

BPI-SF baseline values are reported in Supplementary Table 6. PAB patients scored significantly higher on all baseline BPI-SF subscales compared to no-PAB patients (*p* < 0.001). The percentage of patients experiencing a complete pain response for the duration of Ra-233 treatment was 31.4% (Table 2). Changes in time of the BPI-SF Worst pain and Average pain subscales are displayed in Fig. 1A, B, respectively, and the other BPI-SF subscales in Supplementary Fig. 3. During treatment, 49.5% of the evaluable sample had a clinically meaningful improvement of the BPI-SF Worst pain subscale (Table 2 and Fig. 1B). Median and mean times to deterioration of the BPI-SF subscales are reported in Table 2 and Fig. 1A. PAB patients had a significantly longer median time to deterioration of the BPI-SF subscale Average pain than no-PAB patients. (Table 2 and Supplementary Fig. 4). PAB patients also had a significantly longer TPP than no-PAB patients (Table 2 and Fig. 1A).

**Table 2:** Patient-reported outcomes: median time to BPI-SF and FACT-P deterioration and pain response.

Outcome variables		Median [IQR], no. of patients (%) [IQR or 95% CI]			*p*^a^
		Evaluable sample (*n* = 105)	Pain at baseline (*n* = 45)	No pain at baseline (*n* = 60)	
Time to BPI-SF deterioration, months					
Worst pain/time to pain progression					0.001
	Median	5.6 [4.7–9]	11.1 [7.6–NR]	4.1 [3.6–5.7]	
	Mean	7.9 [6.4–9.4]	11.2 [8.5–13.8]	6.1 [4.6–7.7]	
Least pain					ns
	Median	7.1 [6.2–NR]	14.1 [6.9–NR]	6.5 [5.8–NR]	
	Mean	10.7 [8.5–12.9]	11.5 [8.3–14.7]	9.6 [7.3–11.9]	
Average pain					0.03
	Median	6.1 [5.5–NR]	12.6 [6.2–NR]	5.5 [4.1–6.8]	
	Mean	9.4 [7.8–11]	11.5 [8.8–14.2]	8 [6.1–9.8]	
Pain now					ns
	Median	6.2 [4.7–NR]	NR [10–NR]	5.7 [4.1–7.2]	
	Mean	9 [7.3–10.6]	11.9 [9.1–14.6]	7.7 [5.8–9.6]	
Overall pain interference					ns
	Median	8.3 [6.5–13.5]	10.6 [7.2–NR]	6.7 [5.7–NR]	
	Mean	10.4 [8.2–12.5]	9.9 [7.1–12.8]	9.8 [7.5–12.1]	
Clinically meaningful improvement of BPI-SF Worst pain during treatment, no. of patients (%)		52 (49.5)	35 (77.7)	17 (28.3)	<0.0001
Pain response, no. of patients (%)					0.004
Complete		33 (31.4)	9 (20.0)	24 (40.0)	0.03
Partial		28 (26.7)	21 (46.7)	7 (11.7)	0.0001
Indeterminate		35 (33.3)	11 (24.4)	24 (40.0)	ns
Progressive pain		6 (5.7)	3 (6.7)	3 (5.0)	ns
Not evaluable		3 (2.8)	1 (2.2)	2 (1.7)	
Time to FACT-P deterioration, months					
Total					ns
	Median	5.7 [3.3–NR]	13.7 [2.5–NR]	5.5 [3.1–NR]	
	Mean	7.8 [6.2–9.3]	8.4 [6.4–10.5]	7 [5.4–8.6]	
Prostate cancer subscale					ns
	Median	9.8 [7–NR]	NR [6.4–NR]	9.8 [7–NR]	
	Mean	11.1 [8.9–13.2]	12.4 [9.6–15.2]	9.9 [7.5–12.3]	
Physical well-being					ns
	Median	NR [7.2–NR]	12.6 [6.4–NR]	NR [NR–NR]	
	Mean	12.4 [10.4–14.4]	10.2 [7–13.5]	12.8 [10.7–14.9]	
Social well-being					ns
	Median	13.2 [11.2–NR]	NR [NR–NR]	13.2 [10.4–NR]	
	Mean	13.2 [11.1–15.3]	14.6 [12.3–17]	12.3 [10–14.6]	
Emotional well-being					ns
	Median	NR [NR–NR]	NR [12.6–NR]	NR [NR–NR]	
	Mean	13.6 [12.1–15.2]	14.4 [12–16.8]	13.1 [11.2–15]	
Functional well-being					ns
	Median	NR [12.7–NR]	12.7 [7.6–NR]	NR [NR–NR]	
	Mean	13.9 [12–15.9]	12.4 [9.2–15.6]	14.2 [12.2–16.2]	
Pain					ns
	Median	10.7 [9–NR]	12.6 [12.6–NR]	9 [5.8–NR]	
	Mean	9.6 [7.9–11.3]	11 [8.9–13.1]	8.3 [6.9–9.7]	
Clinically meaningful improvement of Total FACT-P during treatment, no. of patients (%)		33 (31.4)	17 (37.7)	16 (26.7)	ns

Clinically meaningful improvement of Total FACT-P was defined as a minimal change of 10 points from baseline for the Total FACT-P score, 3 points from baseline for the subscales and 2 points from baseline for pain. The Clinically Meaningful improvement of BPI-SF score (CMC-BPI) was defined as a change of score of at least 30% from baseline score, with a minimum of 2 points.

*BPI-SF* Brief Pain Inventory-Short Form, *FACT-P* Functional Assessment of Cancer Therapy-Prostate, *NR* not reached, *ns* not significant.

^a^Pain at baseline vs no-pain at baseline.

#### FACT-P

FACT-P baseline values are reported in Supplementary Table 6. PAB patients had significantly lower baseline Total FACT-P scores than no-PAB patients (95.2 and 107.6, respectively, *p* < 0.001), suggesting a worse HRQoL.

**Fig. 2:**
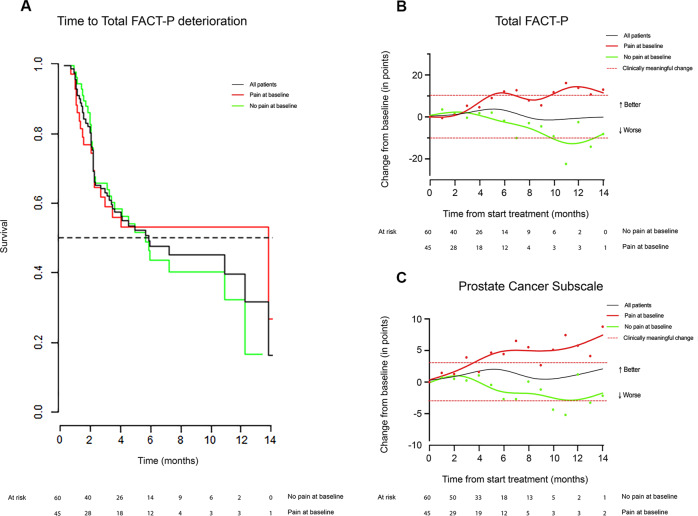
Functional Assessment of Cancer Therapy–Prostate (FACT-P). **A** Kaplan-Meier estimates of time to clinically meaningful Total Functional Assessment of Cancer Therapy–Prostate (FACT-P) score deterioration for the evaluable sample (black line), patients with pain at baseline (red line), and patients without pain at baseline (green line). The horizontal dotted line represents 50% events. **B** Change in Total FACT-P. **C** Prostate cancer subscale scores in time for the evaluable sample (black line), patients with pain at baseline (red line), and patients without pain at baseline (green line). Data points show average score at time points, while the lines are made to fit the trend of change of score in time. The horizontal dotted lines represent the threshold for clinically meaningful change from baseline.

During treatment, 31.4% of the evaluable sample had a clinically meaningful improvement of Total FACT-P, with no significant difference between PAB and no-PAB patients (Table 2 and Fig. 2B). Changes in time of the FACT-P subscales are displayed in Supplementary Fig. 5. Median and mean TTFD and other deteriorations of FACT-P subscales are reported in Table 2. There were no significant differences in deterioration times of Total FACT-P or the other FACT-P subscales between PAB and no-PAB patients. (Table 2, Fig. 2A, and Supplementary Fig. 6).

#### Analgesics use and integration of PROMs results

**Fig. 3:**
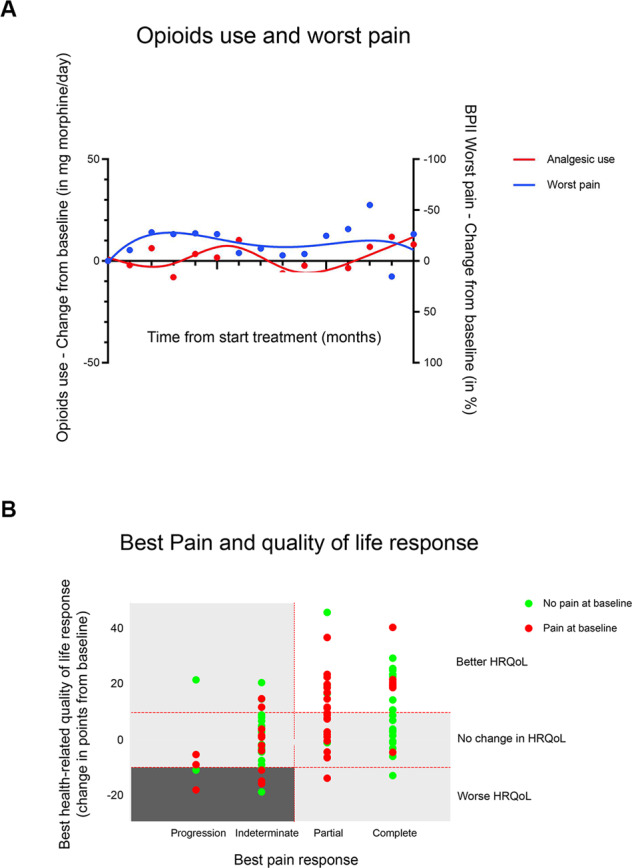
Integrated pain and health-related quality of life response. **A** Percentage change in Brief Pain Inventory Short Form (BPI-SF) – Worst pain subscale scores from baseline in time (blue line) and change in average analgesics use from baseline in mg morphine equivalents per day (red line). **B** Patients were categorized for their best pain response (Worst pain subscale) integrated with opioid drugs use according to IBMCWP recommendations (horizontal axis: progression, indeterminate, partial, and complete response) and for their best health-related quality of life response (vertical axis: Total FACT-P clinically meaningful better or worse or not meeting these criteria and therefore considered as no change). The red, horizontal dotted lines represent the threshold for clinically meaningful Total FACT-P change (10 points), while the vertical dotted line separates progression and indeterminate pain responses from partial and complete pain responses. Red dots represent pain at baseline patients and green dots no-pain at baseline patients.

Use of analgesics in the evaluable sample decreased during Ra-223 treatment and remained low during follow-up (Fig. 3A and Supplementary Fig. 7). The score of the BPI-SF subscale Worst pain did not show a clinically meaningful change during Ra-223 treatment and in follow-up. Ninety-five patients had sufficient data to be categorized for best pain response and Total FACT-P response. Fifty-five (57.9%) had an IOCR, of whom 27 (49.1%) were PAB and 28 (50.9%) were no-PAB patients (Fig. 3B).

## Discussion

In the ALSYMPCA, pain was evaluated using the non-pain-specific questionnaires FACT-P and EQ-5D [5]. Evaluation of opioids use was limited to baseline opioid use and 3 monthly assessments of opioid use in patients without baseline use. A non-significant reduction in pain was found between Ra-223- and placebo-treated patients at 16 and 24 weeks of treatment [4, 5]. The percentages of patients experiencing a clinically meaningful improvement of Total FACT-P in our cohort was comparable to ALSYMPCA (31.4% and 24.6%, respectively) [5]. However, there are critical differences between the ALSYMPCA population and the population in this cohort. The ALSYMPCA was conducted in a time when docetaxel was the only treatment option for mCRPC patients. Consequently, in ALSYMPCA, patients received Ra-223 after docetaxel or as a first-line mCRPC treatment. Contemporary mCRPC patients have multiple treatment options. In this study, more than half of the patients received at least 2 treatments prior to Ra-223 treatment. It can be assumed that the extensively pretreated patients in this study are prone to poorer performance, while strict patient selection might compensate for that. Moreover, in ALSYMPCA patients were symptomatic, while in this study the majority of patients had no-PAB. Unfortunately, baseline Total FACT-P scores of patients included in ALSYMPCA have not been made available [5, 16]. In line with our results, three small retrospective studies, using various measurements, suggested that approximately half of the patients experience reduced pain during Ra-223 treatment [17–19]. One prospective study, using the cancer-specific EORTC-QLQ-C30 measurement, showed no HRQoL deterioration during Ra-223 treatment [20].

In this study, outcomes of the different PROMs were integrated into an IOCR, which was established in 58% of patients. Cancer-related pain and HRQoL are not mutually exclusive, as was reported previously [21, 22]. However, some patients had more pain but a better HRQoL, while others experienced less pain and a worse HRQoL. In part, this can be explained by inclusion of the best pain response and best HRQoL change for establishing the IOCR. Moreover, HRQoL can also be affected by other domains than pain, including fatigue, psychological distress, financial problems, or social problems [23]. Another possible explanation is that this is caused by response shift, where patients accommodate to their pain by cognitive reframing and re-prioritizing of previously held values, internal standards, and expectations to help cope with high levels of pain [24].

The strength of this study lies in the inclusion of a contemporary real-world population, pretreated with multiple mCRPC treatment options. Moreover, both symptomatic and asymptomatic patients were included, as this inclusion criterion of the ALSYMPCA is generally not considered in daily practice. This makes the results of this study directly applicable to current prostate cancer patients’ treatment. There is a growing interest in real-life data, however, PROMs are rarely reported. In line with the increased interest in PROMs outcomes from randomized trials, we would argue in favor of including these outcomes in real-life cohorts.

Limitations of this study include its non-randomized nature and the likelihood of survival and selection bias. Another limitation is the lower than expected questionnaire completion rates. The percentage of patients evaluable was within the previously reported 10–70% range of response rates in studies on self-reported outcome measures in real-life populations [25–27], but lower than the 40% we assumed for the power calculation. It was previously reported that a higher frailty score was a strong predictor for non-completion [28]. The older age and more advanced disease, and with that a presumably higher frailty score of patients in our cohort compared with similar studies in patients with other cancers, might explain the low completion rates. Despite the above, the evaluable sample seemed to be representative for the registry sample since there were no major differences in baseline characteristics.

In conclusion, our study shows that a significant proportion of Ra-223-treated symptomatic and asymptomatic, extensively pretreated mCRPC patients experience an improved HRQoL and pain response. These results suggest that the majority of contemporary mCRPC patients derive clinical benefit from Ra-223 treatment.

## Supplementary information

Supplementary Table 1

Supplementary Table 2

Supplementary Table 3

Supplementary Table 4

Supplementary Table 5

Supplementary Table 6

Supplementary Text 1

Supplementary figure legends

Supplementary Figure 1

Supplementary Figure 2

Supplementary Figure 3

Supplementary Figure 4

Change in Brief Pain Inventory Short Form (BPI-SF) subscale scores over time in the evaluable sample (black line), patients with pain at baseline (red line), and patients without pain at baseline (green line).

Supplementary Figure 6.

Supplementary Figure 7

## Data Availability

The data underlying this article will be shared on reasonable request to the corresponding author.
