# Supplementary material for: Integrated analysis of pain, health-related quality of life, and analgesic use in patients with metastatic castration-resistant prostate cancer treated with Radium-223
Source: Prostate Cancer Prostatic Dis. 2021 Aug 26;25(2):248–55. doi: 10.1038/s41391-021-00412-6 (PMC9184275; doi:10.1038/s41391-021-00412-6)
Supplement: Supplementary file 1 — Supplementary Table 1 [file 41391_2021_412_MOESM1_ESM.docx]

**Supplementary table 1. Questionnaires used to asses health-related quality of life (HRQoL), pain and analgesics use.**

|  | Number of items | Score range | Clinically meaningful change (CMC) | Description | | Completion dates | |  |
| --- | --- | --- | --- | --- | --- | --- | --- | --- |
| Functional Assessment of Cancer Therapy-Prostate (FACT-P) | | | | | A validated questionnaire used to evaluate HRQol in mCRPC patients. A higher FACT-P total score represents better HRQoL | | Baseline, once every 4 weeks until start of subsequent treatment or death | |
| Total score | 39 | 0-156 | 10 points from baseline |  | |  | |  |
| Prostate cancer subscale | 12 | 0-48 | 3 points from baseline |  | |  | |  |
| Physical well-being | 7 | 0-28 | 3 points from baseline |  | |  | |  |
| Functional well-being | 7 | 0-28 | 3 points from baseline |  | |  | |  |
| Emotional well-being | 6 | 0-24 | 3 points from baseline |  | |  | |  |
| Social well-being | 7 | 0-28 | 3 points from baseline |  | |  | |  |
| Pain | 4 | 0-16 | 2 points from baseline |  | |  | |  |
|  | | | | | | |  | |
| Brief Pain Inventory-Short Form (BPI-SF) | | | | | A commonly used validated questionnaire used to evaluate pain in cancer trials. This questionnaire assesses several aspects of pain. Each aspect is assesed with an individual score on a scale of 0-10, with higher scores representing more pain. | | Baseline, once every 4 weeks until start of subsequent treatment or death | |
| Worst pain | 1 | 0-10 | Increase ≥ 30% and ≥ 2 point from baseline |  | |  | |  |
| Least Pain | 1 | 0-10 | Increase ≥ 30% and ≥ 2 point from baseline |  | |  | |  |
| Mean pain | 1 | 0-10 | Increase ≥ 30% and ≥ 2 point from baseline |  | |  | |  |
| Current Pain | 1 | 0-10 | Increase ≥ 30% and ≥ 2 point from baseline |  | |  | |  |
| Pain interference | 7 | 0-10 | Increase ≥ 30% and ≥ 2 point from baseline | Described as pain during daily activities (*e.g.:* sleep, mood) | |  | |  |
|  |  |  |  |  | |  | |  |
| List of opioid drugs used | | | | Free text list of all opioid drugs used in the previous 4 weeks | | Baseline, once every 4 weeks until start of subsequent treatment or death | |  |
