# Supplementary material for: Integrated analysis of pain, health-related quality of life, and analgesic use in patients with metastatic castration-resistant prostate cancer treated with Radium-223
Source: Prostate Cancer Prostatic Dis. 2021 Aug 26;25(2):248–55. doi: 10.1038/s41391-021-00412-6 (PMC9184275; doi:10.1038/s41391-021-00412-6)
Supplement: Supplementary file 2 — Supplementary Table 2 [file 41391_2021_412_MOESM2_ESM.docx]

**Supplementary table 2: Conversion of opioid drugs to oral morphine**

| **Morphine** | | **Fentanyl** | **Oxycodon** | | **Hydromorphine** | | **Tramadol** | **Buprenophine** | **Tapentadol** |
| --- | --- | --- | --- | --- | --- | --- | --- | --- | --- |
| Oral | S.C./I.V | Patch | Oral | S.C./I.V. | Oral | S.C/I.V. | Oral | Patch | Oral |
| Mg/24h | Mg/24h | μg/h | Mg/24h | Mg/24h | Mg/24h | Mg/24h | Mg/24h | μg/h | Mg/24h |
| 30 | 10 | 12 | 20 | 10 | 8 | 2 | 150 | - | 75-100 |
| 60 | 20 | 25 | 40 | 20 | 12 | 4 | 300 | - | 150 |
| 120 | 40 | 50 | 80 | 40 | 24 | 8 | - | 52.2 | 300 |
| 180 | 60 | 75 | 120 | 60 | 36 | 12 | - | - | 450 |
| 240 | 80 | 100 | 160 | 80 | 48 | 16 | - | 105 | - |
| 360 | 120 | 150 | 240 | 120 | 72 | 24 | - | - | - |
| 480 | 160 | 200 | 320 | 160 | 96 | 32 | - | - | - |
