# Supplementary material for: Integrated analysis of pain, health-related quality of life, and analgesic use in patients with metastatic castration-resistant prostate cancer treated with Radium-223
Source: Prostate Cancer Prostatic Dis. 2021 Aug 26;25(2):248–55. doi: 10.1038/s41391-021-00412-6 (PMC9184275; doi:10.1038/s41391-021-00412-6)
Supplement: Supplementary file 3 — Supplementary Table 3 [file 41391_2021_412_MOESM3_ESM.docx]

**Supplementary Table 3:** **Definitions secondary endpoints**

| Time to Total FACT-P Deterioration (TTFD) | time from the date of first Ra-223 course to the first moment of a decrease in Total FACT-P score of at least 10 points from baseline |
| --- | --- |
| Time to Pain Progression (TPP) | time from the date of first Ra-223 treatment to the moment of an increase in worst pain score fulfilling the CMC-FACT criteria |
| Progression Free Survival (PFS) | date of first Ra-223 treatment to the date of confirmed progression |
| Progression | Progression was confirmed at, clinical progression (defined as clinical signs of progression), radiological progression (according to RECIST v. 1.1)†, onset of subsequent treatment or death, all in line with PCWG3 reccomendations††. |
| Overall Survival (OS) | Date of the first Ra-223 cycle to the date of death |

†Eisenhauer EA, Therasse P, Bogaerts J et al. New response evaluation criteria in solid tumours: Revised RECIST guideline (version 1.1). Eur. J. Cancer 2009; 45(2):228–247.

††Scher HI, Morris MJ, Stadler WM et al. Trial Design and Objectives for Castration-Resistant Prostate Cancer: Updated Recommendations From the Prostate Cancer Clinical Trials Working Group 3. J. Clin. Oncol. 2016; 34(12):1402–18.
