# Supplementary material for: Integrated analysis of pain, health-related quality of life, and analgesic use in patients with metastatic castration-resistant prostate cancer treated with Radium-223
Source: Prostate Cancer Prostatic Dis. 2021 Aug 26;25(2):248–55. doi: 10.1038/s41391-021-00412-6 (PMC9184275; doi:10.1038/s41391-021-00412-6)
Supplement: Supplementary file 4 — Supplementary Table 4 [file 41391_2021_412_MOESM4_ESM.docx]

**Supplementary table 4: Comparison of treatment outcomes of the registry sample and the evaluable sample for pain and quality of life**

|  | | **Median [IQR], Number of Patients (%) or value (n=patients evaluable)** | |  |
| --- | --- | --- | --- | --- |
|  | | Registry sample (*n=300*)* | Evaluable sample (*n=105*) | *P* |
| **Treatment outcomes** | | |  |  |
| Follow-up, months | | 13.2 (12.1-14.4) | 13.2 (11.4-15) | Ns |
| No. of Radium-223 cycles | |  |  | Ns |
|  | Median no. of cycles | 5.0 [3.0-6.0] | 5 [4-6] |  |
| ALP decline | | *(n=255)* | *(n=102)* |  |
|  | ≥30% | 122 (47.8) | 39 (37%) | Ns |
|  | ≥50% | 56 (22.0) | 18 (17%) | Ns |
|  | ≥90% | 1 (0.4) | 1 (1%) | Ns |
| Time to ALP progression, months | |  |  | Ns |
|  | Median | 6.7 (6.4 – 7.4) | 6.8 (6.2-NR) |  |
|  | Mean | 7.9 (6.7 – 9.2)) | 8.0 (6.7-9.2) |  |
| PSA decline | | *n=256* | *n=103* |  |
|  | ≥30% | 16 (6.3) | 7 (7%) | Ns |
|  | ≥50% | 11 (4.3) | 2 (2%) | Ns |
|  | ≥90% | 3 (1.2) | 2 (1.8%) | Ns |
| Time to first SSE, months | | Median not reached | Median not reached |  |
| Progression free survival, months | | 5.1 (4.5-5.8) | 5.2 (4.8-6) | Ns |
| Overall Survival, months | | 15.2 (12.8-17.6) | 19.6 (16.6-NR) | 0.04 |
| Time to subsequent treatment, months | | 5.9 (4.1-7.7) | 3.7 (2.7-8.8) | Ns |
| Hospital admission during Radium-223 treatment | | 82 (28.1) | 24 (23%) | Ns |

**Legend:**

ECOG: Eastern Cooperative Oncology Group; PSA: serum Prostate Specific Antigen; ALP: serum Alkaline Phosphatase; LDH: Lactate Dehydrogenase; SSE: Symptomatic Skeletal Event. Ns:Not significant; Base-line characteristics of the whole population was previously described.^16^
