# Supplementary material for: Integrated analysis of pain, health-related quality of life, and analgesic use in patients with metastatic castration-resistant prostate cancer treated with Radium-223
Source: Prostate Cancer Prostatic Dis. 2021 Aug 26;25(2):248–55. doi: 10.1038/s41391-021-00412-6 (PMC9184275; doi:10.1038/s41391-021-00412-6)
Supplement: Supplementary file 5 — Supplementary Table 5 [file 41391_2021_412_MOESM5_ESM.docx]

**Supplementary table 5**: **Completion rates for questionnaires**

| Radium cycle | | Number of patients | At least one completed questionnaire (%) | All three questionnaires completed (%) | All three questionnaires completed including baseline (%) |
| --- | --- | --- | --- | --- | --- |
|  | Baseline | 300 | 126 (42) | 121 (40) | 121 (40) |
|  | Cycle 1 | 290 | 184 (63) | 181 (62) | 66 (23) |
|  | Cycle 2 | 272 | 182 (67) | 181 (67) | 80 (29) |
|  | Cycle 3 | 250 | 170 (68) | 168 (67) | 74 (30) |
|  | Cycle 4 | 210 | 130 (62) | 130 (62) | 58 (28) |
|  | Cycle 5 | 164 | 110 (67) | 109 (67) | 55 (34) |
|  | Cycle 6 | 140 | 108 (77) | 108 (77) | 46 (33) |
