# Supplementary material for: Integrated analysis of pain, health-related quality of life, and analgesic use in patients with metastatic castration-resistant prostate cancer treated with Radium-223
Source: Prostate Cancer Prostatic Dis. 2021 Aug 26;25(2):248–55. doi: 10.1038/s41391-021-00412-6 (PMC9184275; doi:10.1038/s41391-021-00412-6)
Supplement: Supplementary file 6 — Supplementary Table 6 [file 41391_2021_412_MOESM6_ESM.docx]

**Supplementary Table 6: Baseline scores of Patient Reported Outcomes**

| Outcome variables | | Mean (SD) | | |  |
| --- | --- | --- | --- | --- | --- |
|  | | Evaluable sample (*n*=105) | Pain at baseline (*n*=45) | No pain at baseline (*n*=60) | *P** |
| BPI-SF | | |  |  |  |
|  | Worst pain | 4.2 (2.8) | 7.0 (1.1) | 2.1 (1.4) |  |
|  | Least pain | 1.8 (1.7) | 2.8(1.8) | 1.1 (1.1) | <0.001 |
|  | Mean pain | 3.1 (2.1) | 5.0 (1.5) | 1.6 (1.2) | <0.001 |
|  | Pain now | 2.4 (2.3) | 4.0 (2.4) | 1.2 (1.3) | <0.001 |
|  | Overall pain interference | 3.0 (2.2) | 4.1 (1.9) | 2.2 (2.0) | <0.001 |
| FACT-P | |  |  |  |  |
|  | Total score | 102.0 (17.4) | 95.2 (13.0) | 107.6 (18.6) | <0.001 |
|  | Prostate cancer subscale | 29.2 (6.6) | 26.2 (4.5) | 31.6 (6.9) | <0.001 |
|  | Physical well- being | 21.1 (4.3) | 19.8 (3.3) | 22.0 (4.7) | <0.001 |
|  | Social well-being | 21.0 (4.4) | 20.5 (4.5) | 21.5 (4.3) | 0.59 |
|  | Emotional well-being | 13.4 (3.5) | 12.5 (3.5) | 14.1 (3.4) | 0.031 |
|  | Functional well-being | 17.5 (5.2) | 16.5 (4.1) | 18.2 (5.8) | 0.039 |
|  | Pain | 9.1 (4.1) | 5.9 (2.7) | 11.5 (3.3) | <0.001 |

**Pain at baseline vs no pain at baseline*

SD: Standard deviation ; BPI-SF: Brief Pain Inventory-Short Form; FACT-P: Functional Assessment of Cancer Therapy-Prostate;
