# Supplementary material for: Integrated analysis of pain, health-related quality of life, and analgesic use in patients with metastatic castration-resistant prostate cancer treated with Radium-223
Source: Prostate Cancer Prostatic Dis. 2021 Aug 26;25(2):248–55. doi: 10.1038/s41391-021-00412-6 (PMC9184275; doi:10.1038/s41391-021-00412-6)
Supplement: Supplementary file 7 — Supplementary Text 1 [file 41391_2021_412_MOESM7_ESM.docx]

**Supplementary Text 1: Sample size calculation**

The sample size was chosen to ensure enough power to detect a meaningful increase (compared to historical placebo) of the number of Ra-223 treated patients with a complete pain response. From the placebo arm of the ALSYMPCA we estimated that, without treatment, up to 20% of patients will have a complete or partial pain response.(1) Our interest is in the power to find a one-sided 95% confidence interval around our observed pain response rate that lies entirely above the ‘placebo rate’ of 20%, under the assumption of a true pain response rate of 30% or more. We computed this power under various assumptions on the percentage of patients returning at least two PROMs forms. Of the scenarios presented in the table below, we considered the number of 120 evaluable patients the most realistic. We estimated a 40% response rate based on the reported 10-70% response rates in previous studies on PROM in real-life populations.(2–4) As shown in the table below, the power at this percentage of evaluable patients is 81%.

**Table: power calculations**

| Number of evaluable patients (out of 300) | 300 | 200 | 150 | 120 | 100 | 50 |
| --- | --- | --- | --- | --- | --- | --- |
| Power to find significant increase in proportion of pain responses compared to 20% | 99% | 95% | 88% | 81% | 70% | 43% |

1. Parker C, Nilsson S, Heinrich D, Helle SI, O’Sullivan JM, Fosså SD, et al. Alpha Emitter Radium-223 and Survival in Metastatic Prostate Cancer. N Engl J Med [Internet]. 2013 [cited 2014 Jul 2];369(3):213–23. Available from: http://www.nejm.org/doi/abs/10.1056/NEJMoa1213755

2. Baruch Y, Holtom BC. Survey response rate levels and trends in organizational research. Hum Relations [Internet]. 2008 Aug [cited 2019 Dec 12];61(8):1139–60. Available from: http://journals.sagepub.com/doi/10.1177/0018726708094863

3. Husson O, de Rooij BH, Kieffer J, Oerlemans S, Mols F, Aaronson NK, et al. The EORTC QLQ-C30 Summary Score as Prognostic Factor for Survival of Patients with Cancer in the “Real-World”: Results from the Population-Based PROFILES Registry. Oncologist [Internet]. 2019 Oct 31 [cited 2019 Dec 12]; Available from: http://www.ncbi.nlm.nih.gov/pubmed/31672773

4. Sinclair M, Otoole J, Malawaraarachchi M, Leder K. Comparison of response rates and cost-effectiveness for a community-based survey: Postal, internet and telephone modes with generic or personalised recruitment approaches. BMC Med Res Methodol. 2012;12.
