# Supplementary material for: Integrated analysis of pain, health-related quality of life, and analgesic use in patients with metastatic castration-resistant prostate cancer treated with Radium-223
Source: Prostate Cancer Prostatic Dis. 2021 Aug 26;25(2):248–55. doi: 10.1038/s41391-021-00412-6 (PMC9184275; doi:10.1038/s41391-021-00412-6)
Supplement: Supplementary file 8 — Supplementary figure legends [file 41391_2021_412_MOESM8_ESM.docx]

**Legend Supplementary figures**

**Supplementary figure 1.** Consort Diagram

**Supplementary figure 2a**. Kaplan-Meier estimate of Overall Survival of the evaluable population

**Supplementary figure 2b.** Kaplan-Meier estimate of Progression Free Survival of the evaluable population

**Supplementary Figure 3.** Kaplan-Meier estimates of Brief Pain Inventory subscales

Kaplan-Meier estimates of time to clinically meaningful BPI–SF subscale scores deterioration for the evaluable sample (black line), patients with pain at baseline (red line) and patients without pain at baseline (green line). The horizontal dotted line represents 50% events

**Supplementary figure 4.** Change in different Brief Pain Inventory subscales over time

Change in Brief Pain Inventory Short Form (BPI-SF) subscales scores over time in the evaluable sample (black line), patients with pain at baseline (red line) and patients without pain at baseline (green line). Data points show average scores at time points, while the lines are made to fit the trend of change of score in time. The horizontal dotted lines represent the threshold for clinically meaningful change from baseline.

**Supplementary figure 5.** Kaplan-Meier estimates of Functional Assessment of Cancer Therapy–Prostate (FACT-P) subscales

Kaplan-Meier estimates of time to clinically meaningful Functional Assessment of Cancer Therapy–Prostate (FACT-P) subscale scores deterioration for the evaluable sample (black line), patients with pain at baseline (red line) and patients without pain at baseline (green line). The horizontal dotted line represents 50% events

**Supplementary figure 6.** Change in different subscales of Functional Assessment of Cancer Therapy–Prostate (FACT-P) scores over time

Change in FACT-P subscale scores in time for the evaluable sample (black line), patients with pain at baseline (red line) and patients without pain at baseline (green line). Data points show average score at time points, while the lines are made to fit the trend of change of score in time. The horizontal dotted lines represent the threshold for clinically meaningful change from baseline.

**Supplementary figure 7.** Change in Opioid use over time

Change in average analgesics use from baseline in mg morphine equivalents per day for the evaluable sample (black line), patients with pain at baseline (red line) and patients without pain at baseline (green line).
