# Supplementary figures and images for: Integrated analysis of pain, health-related quality of life, and analgesic use in patients with metastatic castration-resistant prostate cancer treated with Radium-223
Source: Prostate Cancer Prostatic Dis. 2021 Aug 26;25(2):248–55. doi: 10.1038/s41391-021-00412-6 (PMC9184275; doi:10.1038/s41391-021-00412-6)

**Supplementary figure 1. Consort diagram**

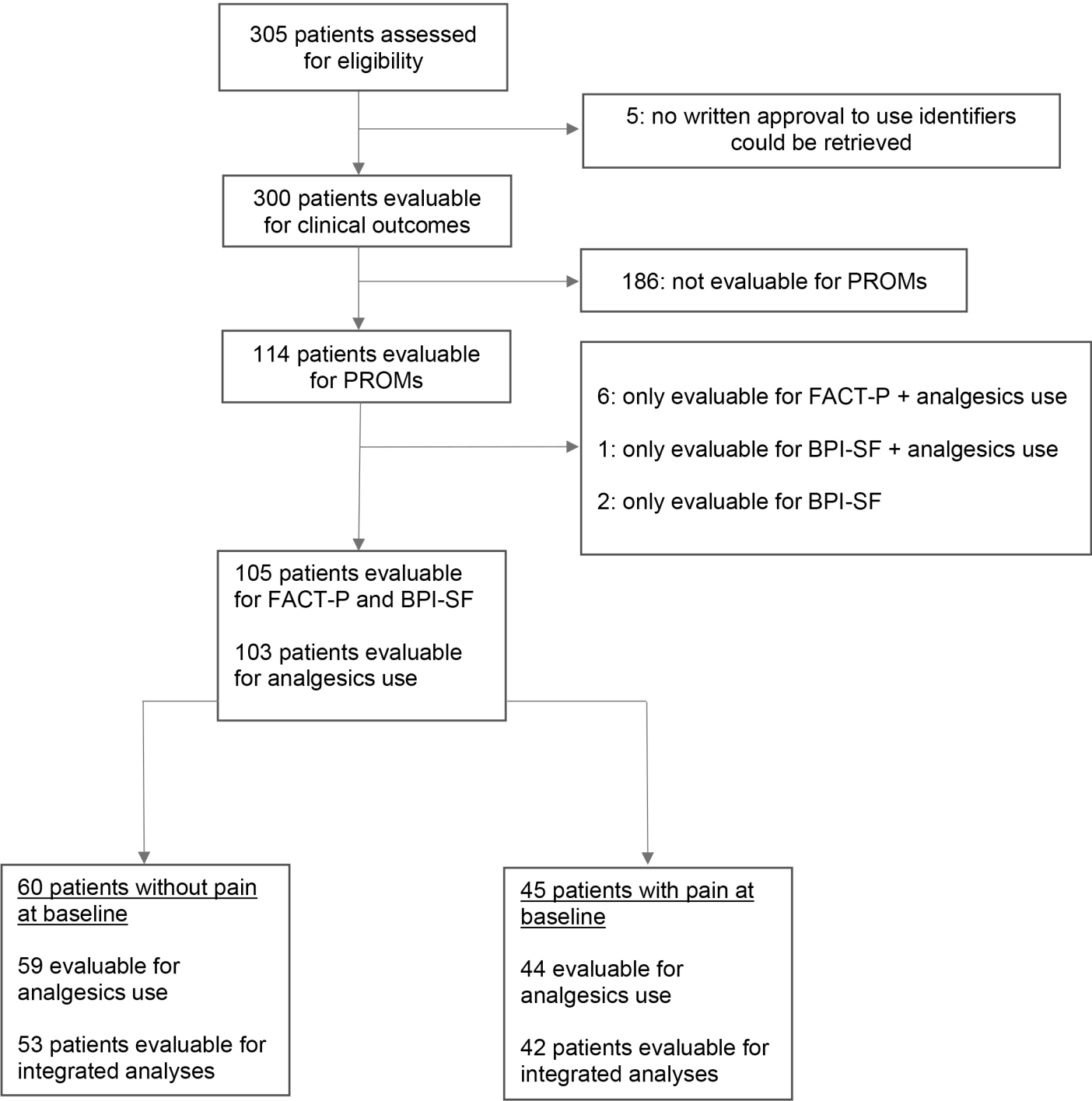

Supplement: Supplementary file 9 — Supplementary Figure 1 [file 41391_2021_412_MOESM9_ESM.pdf]

A

## Overall Survival

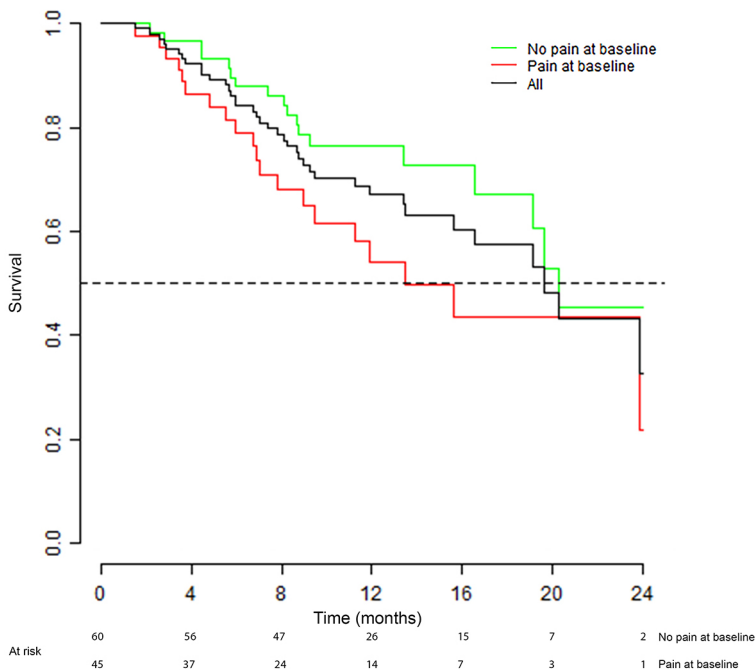

B

## Progression Free Survival

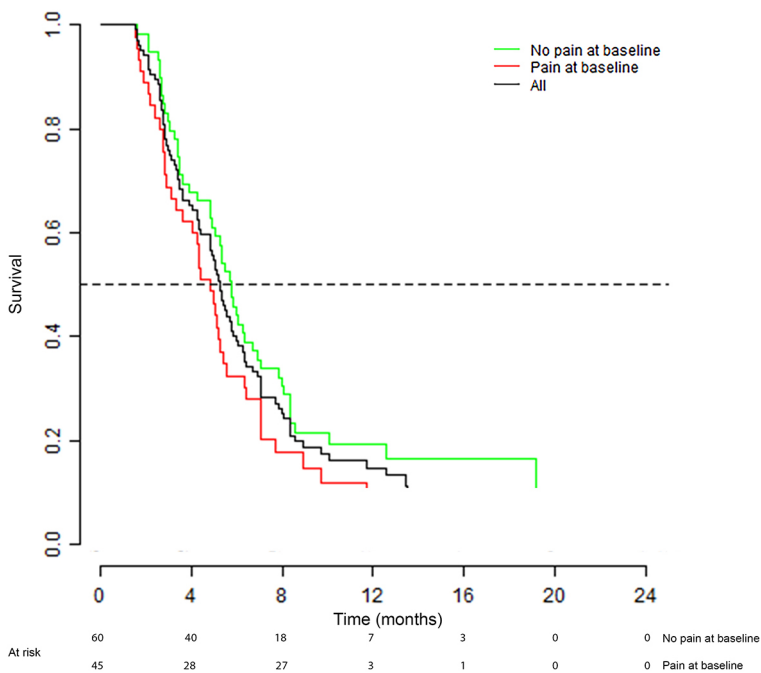

Supplement: Supplementary file 10 — Supplementary Figure 2 [file 41391_2021_412_MOESM10_ESM.pdf]

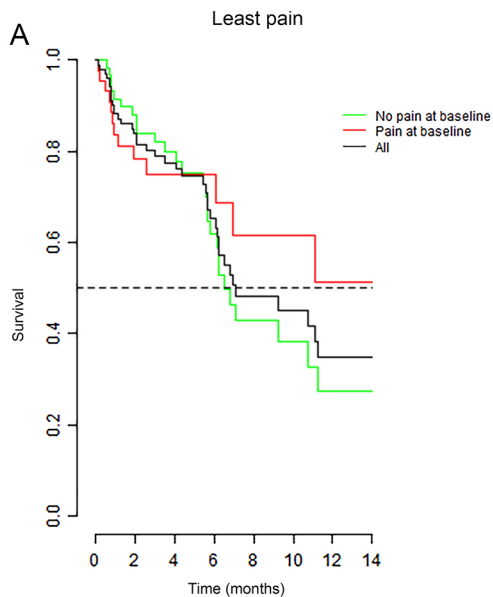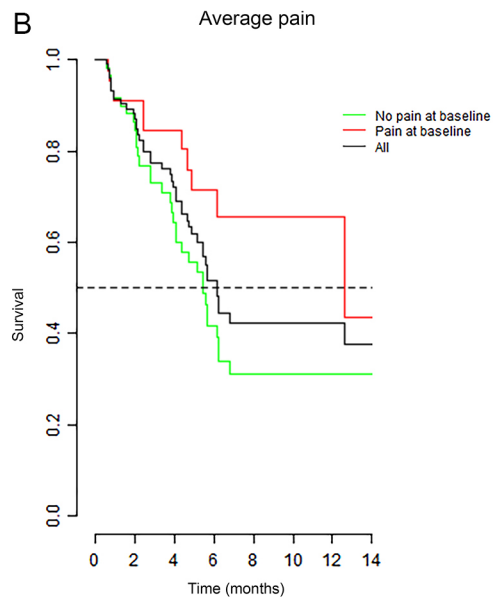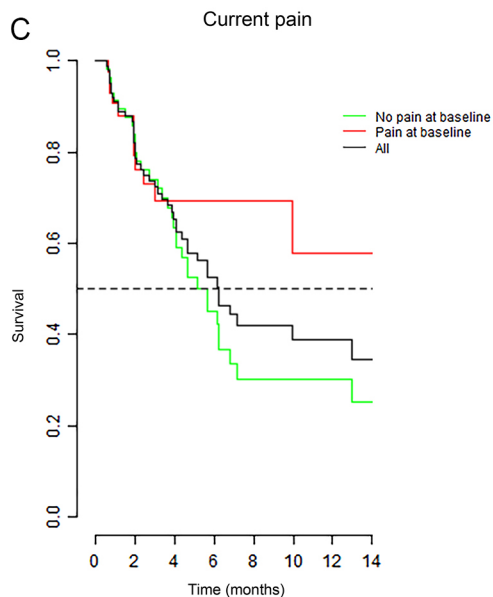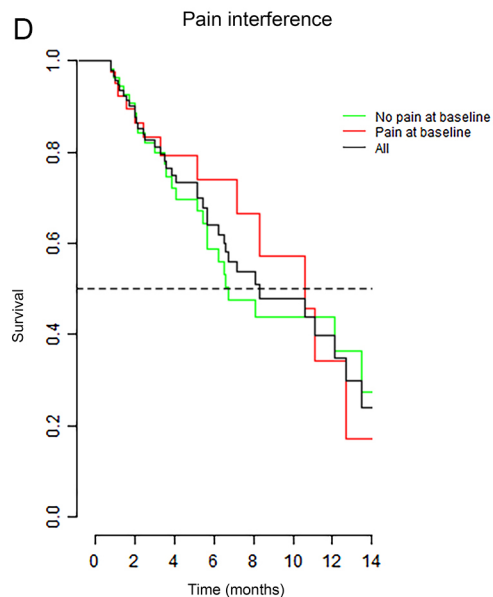

Supplement: Supplementary file 11 — Supplementary Figure 3 [file 41391_2021_412_MOESM11_ESM.pdf]

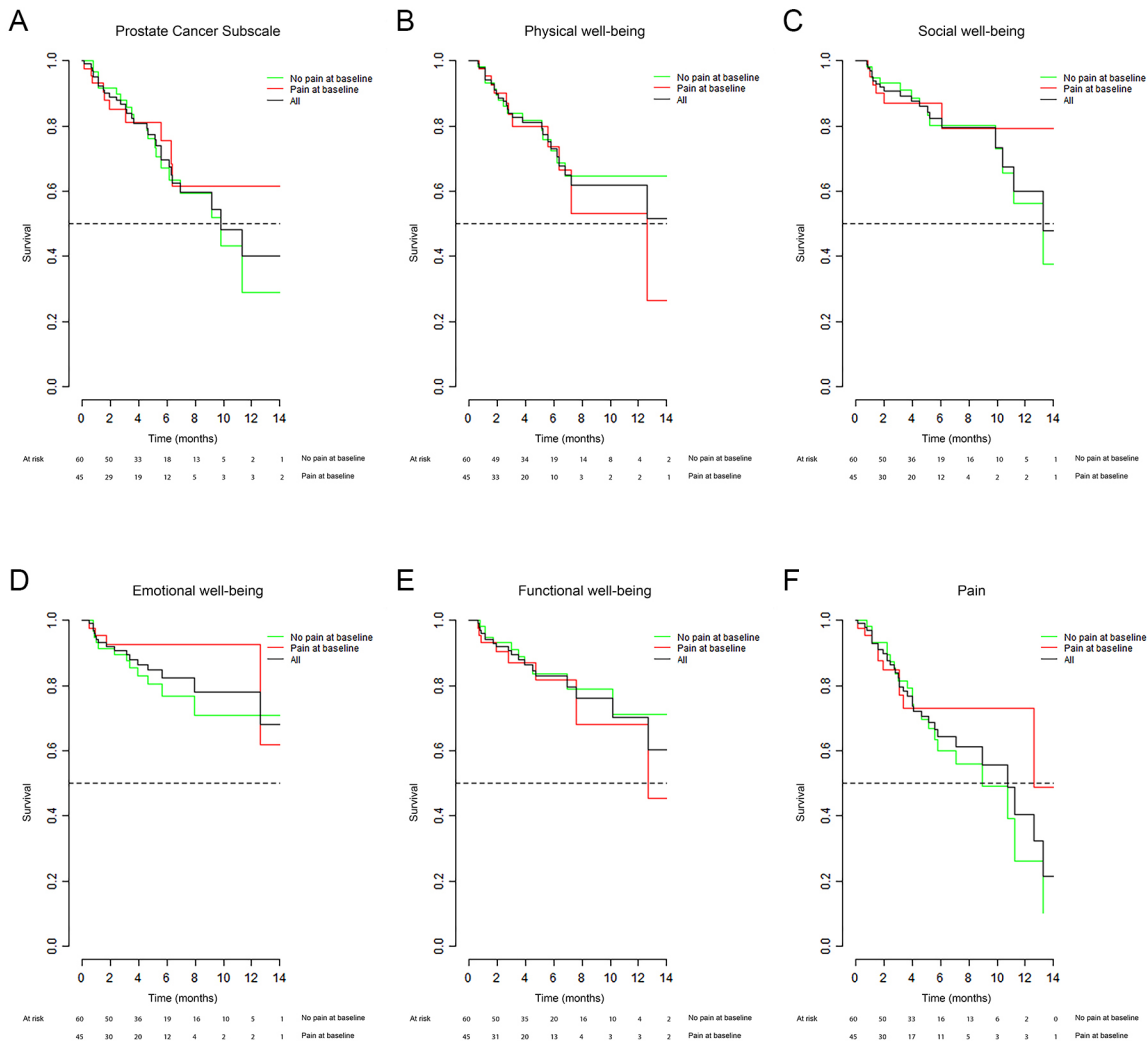

Supplement: Supplementary file 13 — Change in Brief Pain Inventory Short Form (BPI-SF) subscale scores over time in the evaluable sample (black line), patients with pain at baseline (red line), and patients without pain at baseline (green line). [file 41391_2021_412_MOESM13_ESM.pdf]

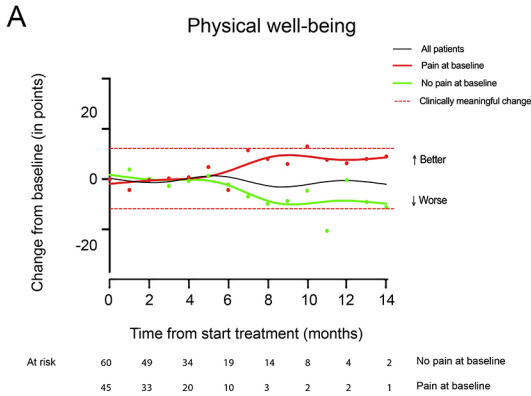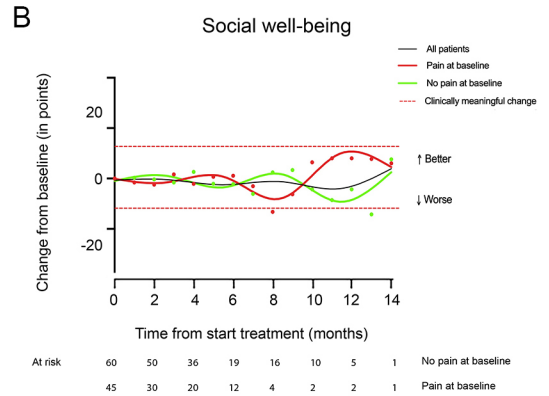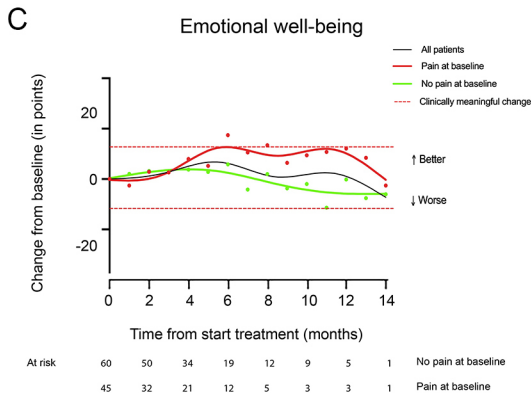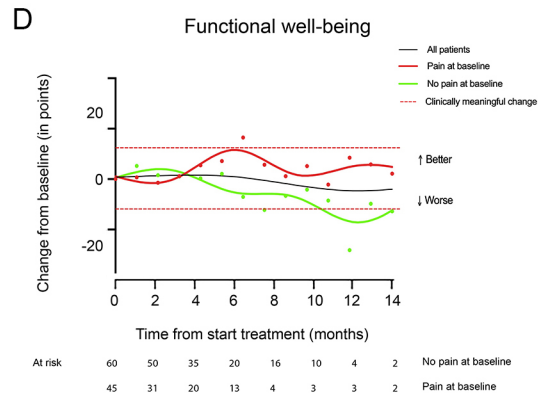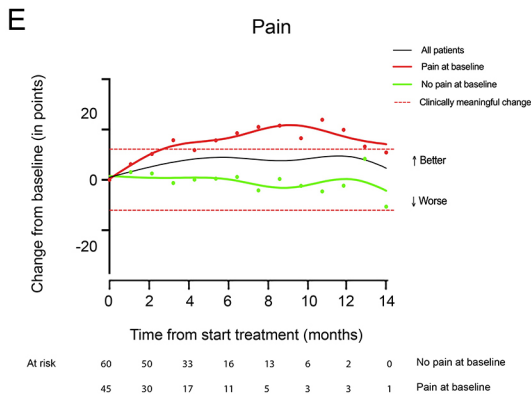

Supplement: Supplementary file 14 — Supplementary Figure 6. [file 41391_2021_412_MOESM14_ESM.pdf]

# Opioids use

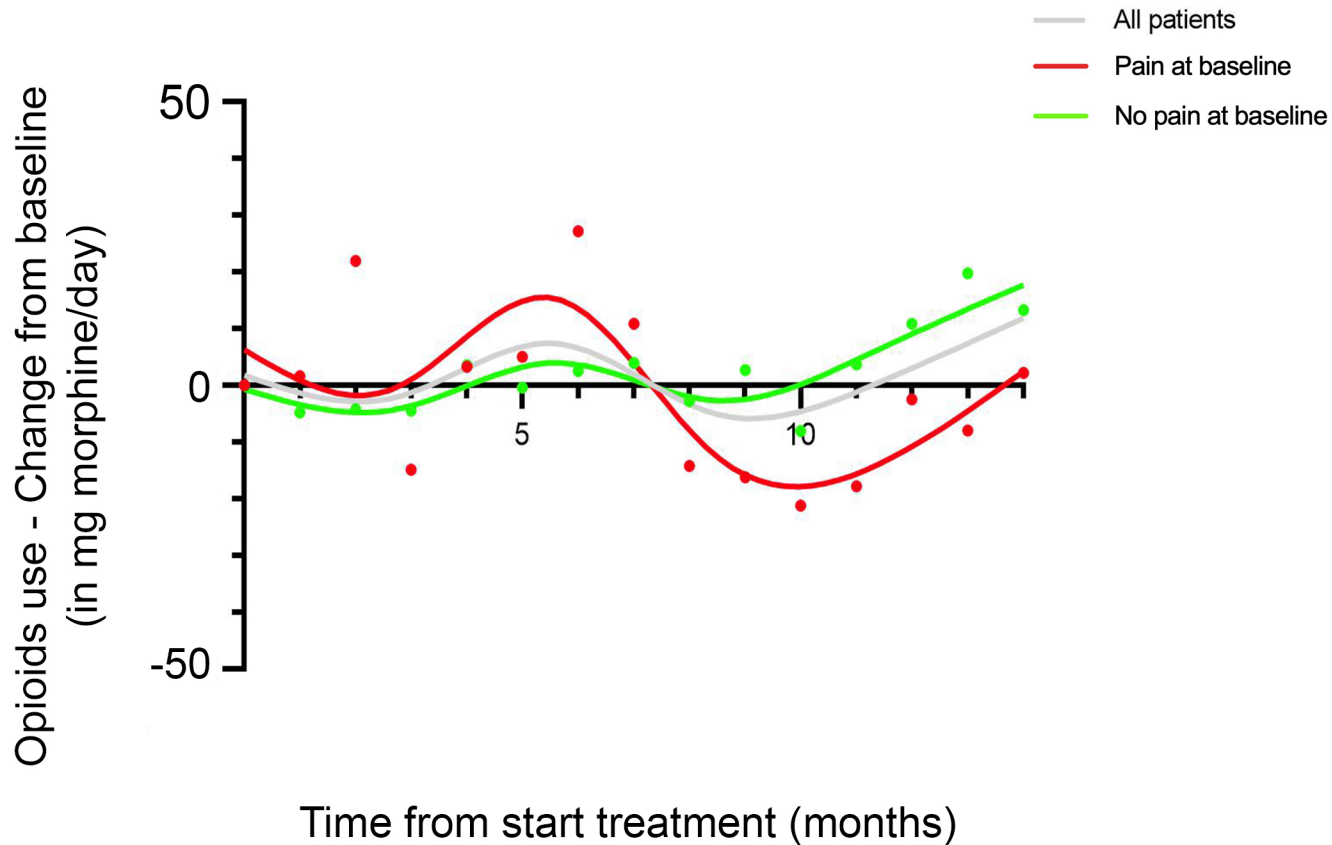

Supplement: Supplementary file 15 — Supplementary Figure 7 [file 41391_2021_412_MOESM15_ESM.pdf]
