# Supplementary material for: Integrated analysis of pain, health-related quality of life, and analgesic use in patients with metastatic castration-resistant prostate cancer treated with Radium-223
Source: Prostate Cancer Prostatic Dis. 2021 Aug 26;25(2):248–55. doi: 10.1038/s41391-021-00412-6 (PMC9184275; doi:10.1038/s41391-021-00412-6)
Supplement: Supplementary file 12 — Supplementary Figure 4 [file 41391_2021_412_MOESM12_ESM.pdf]

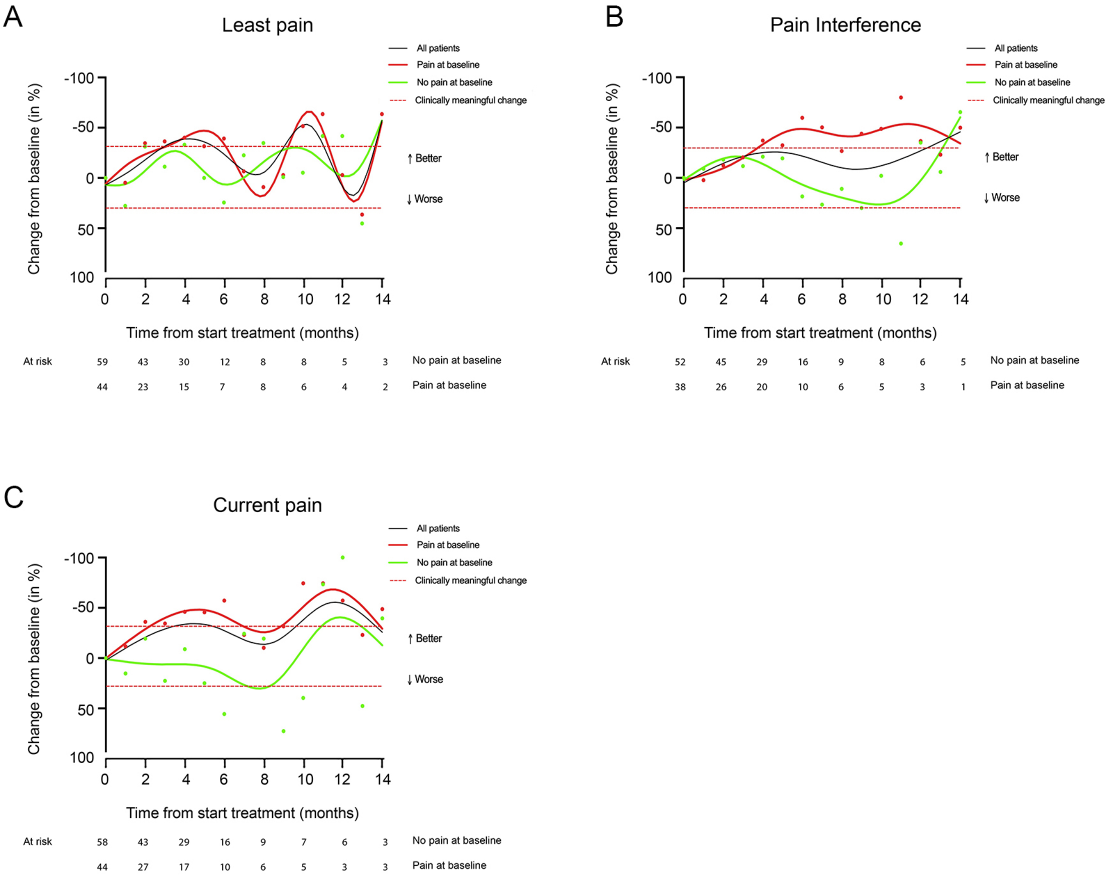
A
Least pain
All patients
Pain at baseline
No pain at baseline
Clinically meaningful change
Change from baseline (in %)
-100
-50
0
50
100
0
2
4
6
8
10
12
14
↑ Better
↓ Worse
Time from start treatment (months)
At risk
59 43 30 12 8 8 5 3 No pain at baseline
44 23 15 7 8 6 4 2 Pain at baseline
B
Pain Interference
All patients
Pain at baseline
No pain at baseline
Clinically meaningful change
Change from baseline (in %)
-100
-50
0
50
100
0
2
4
6
8
10
12
14
↑ Better
↓ Worse
Time from start treatment (months)
At risk
52 45 29 16 9 8 6 5 No pain at baseline
38 26 20 10 6 5 3 1 Pain at baseline
C
Current pain
All patients
Pain at baseline
No pain at baseline
Clinically meaningful change
Change from baseline (in %)
-100
-50
0
50
100
0
2
4
6
8
10
12
14
↑ Better
↓ Worse
Time from start treatment (months)
At risk
58 43 29 16 9 7 6 3 No pain at baseline
44 27 17 10 6 5 3 3 Pain at baseline
